# Modification of 6SV to remove skylight reflected at the air-water interface: Application to atmospheric correction of Landsat 8 OLI imagery in inland waters

**DOI:** 10.1371/journal.pone.0202883

**Published:** 2018-08-24

**Authors:** Zhaoyi Lu, Junsheng Li, Qian Shen, Bing Zhang, Hao Zhang, Fangfang Zhang, Shenglei Wang

**Affiliations:** 1 Key Laboratory of Digital Earth Science, Institute of Remote Sensing and Digital Earth, Chinese Academy of Sciences, Beijing, China; 2 University of Chinese Academy of Sciences, Beijing, China; Mercy Hospital, SIERRA LEONE

## Abstract

During the atmospheric correction of remote sensing data in inland waters, the original Second Simulation of the Satellite Signal in the Solar Spectrum-Vector version (6SV) model does not eliminate the specular reflection of downward skylight radiance at the air-water interface. Thus, we propose a modified version of the 6SV model (M6SV) that does remove reflected skylight at the air-water interface. We apply the new model to the atmospheric correction of a Landsat 8 Operational Land Imager (OLI) image over Taihu Lake, China, where the aerosol optical depth is known. *In situ* reflectance measurements acquired concurrently with the L8/OLI image are used to validate the performance of the new M6SV algorithm. To further analyze the merits and demerits of M6SV, the model is compared with two short-wave infrared (SWIR)-based atmospheric correction models: the Sea-Viewing Wide Field-of-View Sensor Data Analysis System short-wave infrared (SD-SWIR) model and the Vanhellemont & Ruddick short-wave infrared with a per scene fixed aerosol type (VR-SWIR-F) model. Comparisons of results from all three L8/OLI image atmospheric corrections with the *in situ* remote sensing reflectance data show that M6SV produces reliable atmospheric corrections in the green and red spectral bands and is an effective alternative for Landsat 8 OLI atmospheric correction in inland waters.

## Introduction

Satellite remote sensing is a cost-effective way to monitor and quantify optical, biological, and ecological processes and phenomena in inland waters at large and transboundary scales. However, signals reaching a sensor over water contain both the desired water-leaving surface features and undesired atmospheric effects caused by absorption and scattering. Thus, atmospheric correction, the manipulation that can remove such undesired effects from sensor received signals, is a crucial procedure for inland waters quality monitoring [1].

Ocean color sensors, including SeaWiFS (Sea-viewing Wide Field-of-view Sensor, 1997–2003), MODIS (Moderate-Resolution Imaging Spectroradiometer, 1999–present), MERIS (MEdium Resolution Imaging Spectrometer, 2002–2012), COCTS (Chinese Ocean Color and Temperature Scanner, 2002–present), and VIIRS (Visible Infrared Imaging Radiometer Suite, 2011–present) [2], usually have high revisit frequency at the expense of reduced spatial resolution (250 to 1200 m). However, the components and optical properties of inland waters are more complex than those of oceans because they vary with location and season. Therefore, imagery from sensors with higher spatial resolution may be important for quality monitoring of inland waters by quantitative remote sensing [3].

Although designed for monitoring land objects, the Landsat satellite series, which consists of eight sensors in operation since 1972 with 30 m spatial resolutions, has been used for more detailed observation or long-term application effectively in coastal [4–6] and inland waters [7–12]. An operational land imager (OLI) was recently launched on Landsat 8 and has been particularly useful for studying inland waters. With improvements in data quality and extensions in spectral coverage, L8/OLI has been readily adopted for aquatic science applications [13–15].

Atmospheric corrections, including those for skylight reflection off of the water surface, are necessary for ocean color remote sensing. While atmospherically contaminated signals contain the path radiance and the desired land- or water-leaving radiance over both land and water, signals over water also include specular reflection of downward skylight radiance off of the air-water interface, sun glitter reflection, and whitecaps. The effects of sun glitter and whitecaps are generally ignored [16], but atmospheric corrections over water should, at least, consider the elimination of specular reflection at the air-water interface.

The 6SV (Second Simulation of the Satellite Signal in the Solar Spectrum-Vector version) atmospheric correction method is based on a physical radiative transfer model (RTM), which features a reliable, specific physical meaning and better generalization. The RTM requires the input of meteorological parameters acquired at the time of the satellite overpass. The source codes for 6SV are freely available and have been widely applied in atmospheric corrections over land. A number of researchers have used 6SV for atmospheric corrections over water [17–19]; several researchers have also compared 6SV to other atmospheric corrections methods over water [6,11]. The application of 6SV in atmospheric corrections over water does involve some challenges; for instance, the atmospheric coefficients calculated by 6SV are intended for surface reflectance (*R*_*s*_, which is a ratio), not remote sensing reflectance (*R*_*rs*_, unit: sr^-1^) [11]. *R*_*rs*_ is the ratio of water-leaving radiance at the air-water interface to downward irradiance and is commonly used in remote sensing over water. The conversion of *R*_*s*_ to *R*_*rs*_ via division by pi is an approximate calculation and does not consider the elimination of specular reflection at the air-water interface.

In this paper, we modify the inland water atmospheric correction algorithm in 6SV to correct for skylight reflected by the water surface. Also, in order to facilitate the retrieval of the nine visible to short-wavelength infrared spectral bands produced by L8/OLI, a new subroutine simulating the L8/OLI measurements is integrated into the algorithm. To examine the performance and applicability of the modified 6SV algorithm, the correction results are compared with those from Sea-Viewing Wide Field-of-View Sensor Data Analysis System short-wave infrared (SD-SWIR) as well as the Vanhellemont & Ruddick short-wave infrared with a per scene fixed aerosol type (VR-SWIR-F). SD-SWIR is the “standard” atmospheric correction algorithm with glint correction [16, 20]. VR-SWIR-F is the modified “standard” atmospheric correction algorithm [21] for extremely turbid waters but without glint correction[3]. For further validation, the corrected results from all three algorithms are compared with *in situ* reflectance measurements acquired at the time of the satellite overpass.

## Materials and methods

### 2.1 Study area

**Fig 1:**
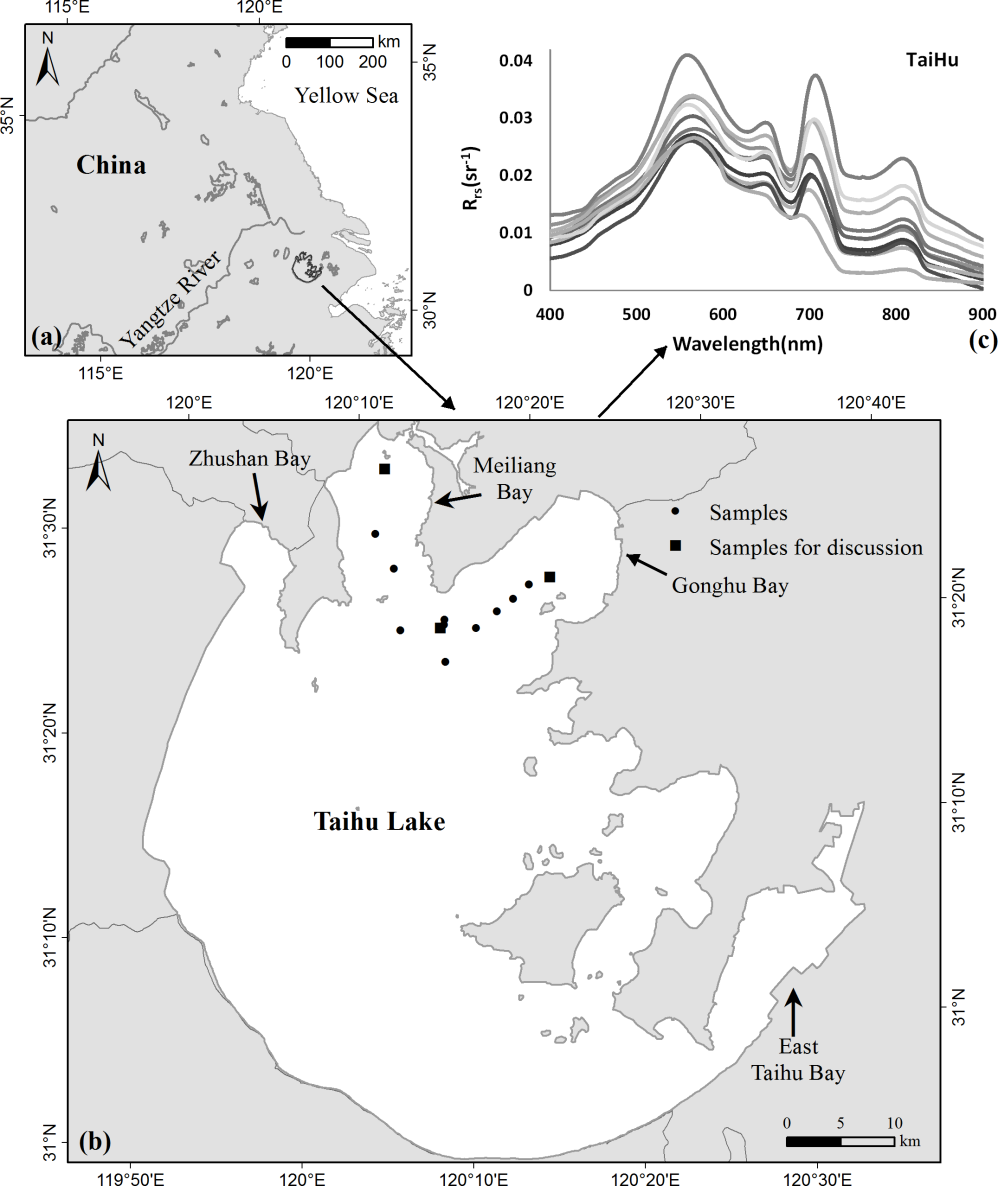
Study site and the *R*_*rs*_ spectra. The geographical locations of (a) Taihu Lake in East China and (b) the *in situ* measurements samples taken on Oct. 26, 2014 at Taihu Lake. (c) The *R*_*rs*_ spectra acquired from the 13 sites. The figure is for illustrative purposes only.

Taihu Lake is located in eastern China (Fig 1(A)) between 119°53′–120°36′ E and 30°56′–31°33′ N, covers 2338 km^2^ of Jiangsu Province and Zhejiang Province, and is surrounded by the cities of Wuxi, Huzhou, Yixing, and Suzhou (Fig 1(B)). Taihu Lake has a mean water depth of 1.9 m [12]. The third-largest inland freshwater lake in China, it supplies water to several million residents in nearby cities and plays critical roles in economic development and the regional ecosystem. Taihu Lake drew global attention after a blue-green algae bloom event in 2007, which highlighted the introduction of regional pollution into its waters. According the measured SD (Secchi depth), which measures 0.3 m on average, Taihu Lake can be regarded as an extremely turbid body of water [22].

### 2.2 *In situ* R_rs_ measurements

Because the L8/OLI satellite passed over Taihu Lake at ~2:31 (UTC), the *in situ* data collection, which covered Gonghu Bay and Meiliang Bay (sites denoted by black dots in Fig 1(B)), was conducted ~2:31 (UTC) over Taihu Lake. The *in situ* data were collected by research team from the Chinese Academy of Sciences for scientific research only, thus no specific permission was required.

The coordinates of the sampling points were determined using a portable GPS and remote sensing reflectance measurements were taken at each station using a FieldSpec® Pro VNIR, ASD spectroradiometer, which covers a spectral range of 350–1050 nm with a spectral resolution of 3 nm. Reflectance was measured using the above-water method [23]. Ten reference plate, water, and skylight spectra were acquired at each station, and the mean spectrum for each type was calculated within ± 5% error to obtain the characteristic remote sensing reflectance (*R*_*rs*_). The formula for *R*_*rs*_ is as follows:

$$R_{\mathrm{rs}}(\lambda )=\frac{L_{w}(\lambda )}{E_{d}\left(0^{+}\right)(\lambda )}=\frac{L_{u}(\lambda )-r_{\mathrm{sky}}L_{\mathrm{sky}}(\lambda )}{\pi L_{p}(\lambda )/\rho_{p}(\lambda )}$$ (1)

where *E*_*d*_(0^+^) is the total surface incident irradiance; *L*_*u*_ *(λ)* denotes the upwelling radiance measured from the water surface; *L*_*sky*_ *(λ)* is the skylight radiance; *L*_*p*_ *(λ)* is the measured reference plate radiance; and *ρ*_*p*_ *(λ)* is the reference plate reflectance, which was about 30%. *r*_*sky*_ is the surface Fresnel contribution, which was interpolated from the lookup tables created by Mobley from measured angles and wind speeds [24–25]. Fig 1(C) shows the *R*_*rs*_ spectra acquired from Taihu Lake on October 26, 2014.

The aerosol optical depth at 550 nm (AOD550) was measured using a hand-held MICROTOPS II Sun photometer linked to a hand-held GPS; this sun photometer measures direct solar radiation in discrete bands selected from five possible channels (440, 675, 870, 936, and 1020 nm) [26]. Ten sets of AOD measurements were acquired in each band in succession at each station. The β and α (described below) of each set were fitted within ± 3% error using Eq (2) [27]. AOD550 values were then calculated, and the mean value was taken.

$$\tau_{a}(\lambda )=\beta \lambda^{-\alpha }$$ (2)

where 0τ_*a*_(*λ*) is the AOD at wavelength *λ*, *β* is the turbidity coefficient, and α denotes the Ångström exponent.

### 2.3 L8/OLI data and *in situ* data matching

The L8/OLI is a push-broom scanner with a swath width of 185 km that covers nine spectral bands. It has eight multispectral bands with 30 m spatial resolution and one pan-chromatic band with 15 m spatial resolution. The central wavelengths of the nine bands are 443, 483, 561, 655, 865, 1609, 2201, 591, and 1373 nm. Compared with the L7/ETM+ (enhanced thematic mapper plus) carried on the Landsat 7 mission, L8/OLI has two additional bands (at 443 and 1373 nm) with narrowed original spectral bands. Because of the longer integration times used in the push-broom scanner, L8/OLI offers SNRs (signal-to-noise ratios) approximately three times higher than those produced by the L7/ETM+ [28]. Furthermore, L8/OLI has better quantization, using 12 instead of 8 bits for radiometric digitization [29].

To evaluate the performance of the M6SV model in removing skylight effects from L8/OLI imagery, we used the model for atmospheric corrections over Taihu Lake. The selected L8/OLI image was acquired at 2:31 (UTC) on October 26, 2014 over Taihu Lake. In order to minimize the effects of temporal and spatial mismatches between satellite and *in situ* data, the time window was narrowed to ~± 1 h of the Landsat 8 overpass. The *in situ* data collection, which involved 13 sampling stations (denoted by black dots in Fig 1(B)), was conducted from 01:11 to 03:53 (UTC) over Taihu Lake on October 26, 2014. In order to ensure spatial data consistency, model-measured *R*_*rs*_ spectral data were derived by averaging a 1 × 1 pixel area (with ~0.03 km spatial resolution) surrounding the *in situ* data location.

### 2.4 Removing skylight reflectance using the modified 6SV model

The 6SV model is an extended version of 6S that takes into consideration light polarization during the signal transfer process [30]. In order to produce atmospheric corrections, 6SV requires inputs such as the meteorological parameters measured at the time of the satellite overpass; the model outputs the atmospheric correction coefficients *x*_*a*_, *x*_*b*_, and *x*_*c*_ [31]. Using these three values, the surface reflectance (*R*_*s*_) can be calculated as follows:

$$R_{s}(\lambda )=\frac{x_{a}(\lambda )L_{t}(\lambda )-x_{b}(\lambda )}{\left(1.0+x_{c}(\lambda )(x_{a}(\lambda )L_{t}(\lambda )-x_{b}(\lambda ))\right)}$$ (3)

where *λ* is the wavelength and *L*_*t*_ is the top-of-the-atmosphere (TOA) radiance received by a satellite sensor in units of W/(m^2^·sr·μm).

The originally published 6SV version did not consider the elimination of specular reflection of downward skylight radiance at the air-water interface; 6SV retrieves the surface reflectance (*R*_*s*_) rather than the remote sensing reflectance (*R*_*rs*_, which has units of sr^-1^); these reflectance values are expressed in Eq (4) and Eq (1), respectively:

$$R_{s}(\lambda )=\frac{L_{t}(\lambda )}{E_{d}\left(0^{+}\right)(\lambda )/\pi }$$ (4)

Thus, we analyzed the successive orders of scattering (SOS) algorithm with the water signal simulation, modified the multiple scattering calculation process in the 6SV source code, and proposed a modified 6SV (M6SV) model to directly generate outputs including the forward scattering radiance (i.e., the downward sky radiance).

If reflections from sun glitter and whitecaps are omitted, the *L*_*t*_ over water received by a satellite sensor is mainly composed of the upward path radiance (*L*_*p*_ (θ_*v–*_)), the specular reflection downward skylight radiance on the water surface (*L*_*spec*_), and the desired *tL*_*w*_ as follows:

$$L_{t}(\lambda )=L_{p}(\theta_{v-},\lambda )+L_{spec}(\lambda )+tL_{w}(\lambda )$$ (5)

where *t* is the diffuse transmittance of the atmosphere; θ_*v−*_is the zenith angle for the sensor; and “–” indicates the upward path direction. The 6SV model does not directly calculate *L*_*spec*_, but does give *L*_*p*_ (θ_*v–*_), from which *L*_*spec*_ can be calculated as follows [32]:

$$L_{\mathrm{spec}}(\lambda )=L_{p}(\theta_{v+},\lambda )r(\theta_{v})\exp(-\tau /\cos\theta_{v})+L_{p}(\theta_{v+},\lambda )r(\theta_{s})\exp(-\tau /\cos\theta_{s})$$ (6)

where *L*_*spec*_ includes two parts: *L*_*spec_1*_*(λ) = L*_*p*_*(*θ_*v+*_, *λ)r(*θ_*v*_*)exp(-*τ*/cos*θ_*v*_*)* and *L*_*spec_2*_*(λ) = L*_*p*_*(*θ_*v+*_, *λ)r(*θ_*s*_*)exp(-*τ*/cos*θ_*s*_*)*. In these equations, *L*_*spec_1*_ is specular reflected skylight, which is diffusely transmitted from solar irradiance; *L*_*spec_2*_ is the path radiance scattered from specular reflected directly transmitted solar irradiance; τ is the total optical depth; *r(*θ_*v*_*)* and *r(*θ_*s*_*)* is the surface Fresnel contribution, θ_*v*_ and θ_*s*_ are the zenith angles for the sensor and the sun, respectively; and *L*_*p*_ (θ_*v+*_), which can be calculated by modifying the SOS counting process, refers to forward-scattered radiation from the sun direction θ_*s*_ to the observation direction θ_*v*_.

In 6SV, the path radiance is calculated through the main function-calling subroutines DISCOM, ATMREF, and OSPOL. The optical thickness, atmospheric scattering, and scattering transmittance are computed by DISCOM and ATMREF. OSPOL is the core of the SOS algorithm; its outputs include the normalized radiation field, which includes the upward path radiance *L*_*p*_ (θ_*v–*_). A detailed flowchart and parameters for these subroutines can be found in related documentation [31].

The goal was to derive a water signal simulation output from the 6SV main function while maintaining the source code structure and atmospheric correction parameters. Thus, the OSPOL code was modified to output the downward radiance *L*_*p*_ (θ_*v+*_) at ground level and the upward radiance *L*_*p*_ (θ_*v–*_) at sensor height. Thus, the new path radiance L^*^_p_*(*θ_*v-*_, *λ) = L*_*p*_*(*θ_*v-*_, *λ) + L*_*spec*_*(λ)* recalculated in the ATMREF code replaces the original path radiance *L*_*p*_ (θ_*v–*_, *λ*).

Downward radiance from the bottom of the atmosphere was also added to OSPOL and is calculated by changing the upward radiance from the top of the atmosphere to downward radiance from the bottom of the atmosphere. In the primary scattering radiation calculations, downward and upward radiances for any optical thickness τ are computed as follows [31]:

$$\begin{array}{l} I^{\left(1\right)}(\tau ;\mu ,\varphi )=\frac{\omega_{0}}{4\pi }\pi F_{0}P(\mu ,\varphi ;‑\mu_{0},\varphi_{0})e^{‑\tau /\mu_{0}}\\ I^{\left(1\right)}(\tau ;‑\mu ,\varphi )=\frac{\omega_{0}}{4\pi }\pi F_{0}P(‑\mu ,\varphi ;‑\mu_{0},\varphi_{0})e^{‑\tau /\mu_{0}} \end{array}$$ (7)

where *I* is the first component of the Stokes vector, which describes the radiation intensity; *μ* is the cosine of the zenith angle; +*μ* corresponds to upward radiation; and–*μ* corresponds to downward radiation, where 1 ≥ *μ* > 0. The parameters *ϕ*, *ω*_*0*_, *F*_*0*_, and *P* are, respectively, the cosine of the azimuth angle, the single scattering albedo, the extraterrestrial solar irradiance, and the scattering phase function.

The definitions of the functions for the optical thickness and upward and downward directions are not changed. To introduce incident light from the bottom of the atmosphere, the downward and upward radiances in the primary scattering radiation term above are modified to:

$$\begin{array}{l} I^{\left(1\right)}(\tau ;\mu ,\varphi )=\frac{\omega_{0}}{4\pi }\pi F_{0}P(\mu ,\varphi ;\mu_{0},\varphi_{0})e^{\left(‑(\tau_{1}-\tau )/\mu_{0}\right)}\\ I^{\left(1\right)}(\tau ;‑\mu ,\varphi )=\frac{\omega_{0}}{4\pi }\pi F_{0}P(‑\mu ,\varphi ;\mu_{0},\varphi_{0})e^{\left(‑(\tau_{1}-\tau )/\mu_{0}\right)} \end{array}$$ (8)

where τ_*1*_ is the total optical thickness. Furthermore, *P* conforms to the reciprocal principle via:

$$\begin{array}{l} P(\mu ,\varphi ;\mu_{0},\varphi_{0})=P(‑\mu ,\varphi ;‑\mu_{0},\varphi_{0})\\ P(‑\mu ,\varphi ;\mu_{0},\varphi_{0})=P(\mu ,\varphi ;‑\mu_{0},\varphi_{0}) \end{array}$$ (9)

The modifications detailed above were added to 6SV as a new subroutine and named OSPOLTOT. We also modified ATMREF to ATMREFTOT and DISCOM to DISCOMTOT to obtain the M6SV model. ATMREFTOT outputs the *L*_*p*_ (θ_*v+*_, *λ*) value computed by the subroutine OSPOLTOT, and DISCOMTOT obtains *L*_*p*_ (θ_*v–*_, *λ*) and *L*_*p*_ (θ_*v+*_, *λ*) by calling subroutine ATMREFTOT. In M6SV, the new outputs *x*_*a*_′, *x*_*b*_′, and *x*_*c*_′ are atmospheric correction parameters that can be used to retrieve the normalized water-leaving reflectance, n*ρ*_*ω*_, directly. *R*_*rs*_ can then be obtained through Eq (10) [33]:

$$R_{rs}(\lambda )=n\rho_{\omega }(\lambda )/\pi$$ (10)

### 2.5 Atmospheric corrections using modified 6SV

While the meaning of the model outputs and results are changed by these modifications, the use of the model remains the same. We ran M6SV with synchronous geometrical image and atmospheric parameters to obtain the atmospheric correction parameters *x*_*a*_′, *x*_*b*_′, and *x*_*c*_′. Then, we substituted these correction parameters into the radiometric corrected image, using Eq (11) to obtain the *R*_*rs*_ of each band.

$$R_{\mathrm{rs}}(\lambda )=\frac{x_{a}^{'}(\lambda )L_{t}(\lambda )-x{'}_{b}(\lambda )}{(1.0+x_{c}^{'}(\lambda )(x_{a}^{'}(\lambda )L_{t}(\lambda )-x_{b}^{'}(\lambda )))\pi }$$ (11)

The L8/OLI spectral response function was not included in the original 6SV. For operational efficiency, we added a subroutine containing the spectral response function and modifying the calling command in the main function in M6SV. The spectral response function is resampled at 2.5 nm intervals in the subroutine. The spectral range of each band spans 0.25 to 4 microns; wavelengths falling outside of the effective spectral range are set to zero.

**Fig 2:**
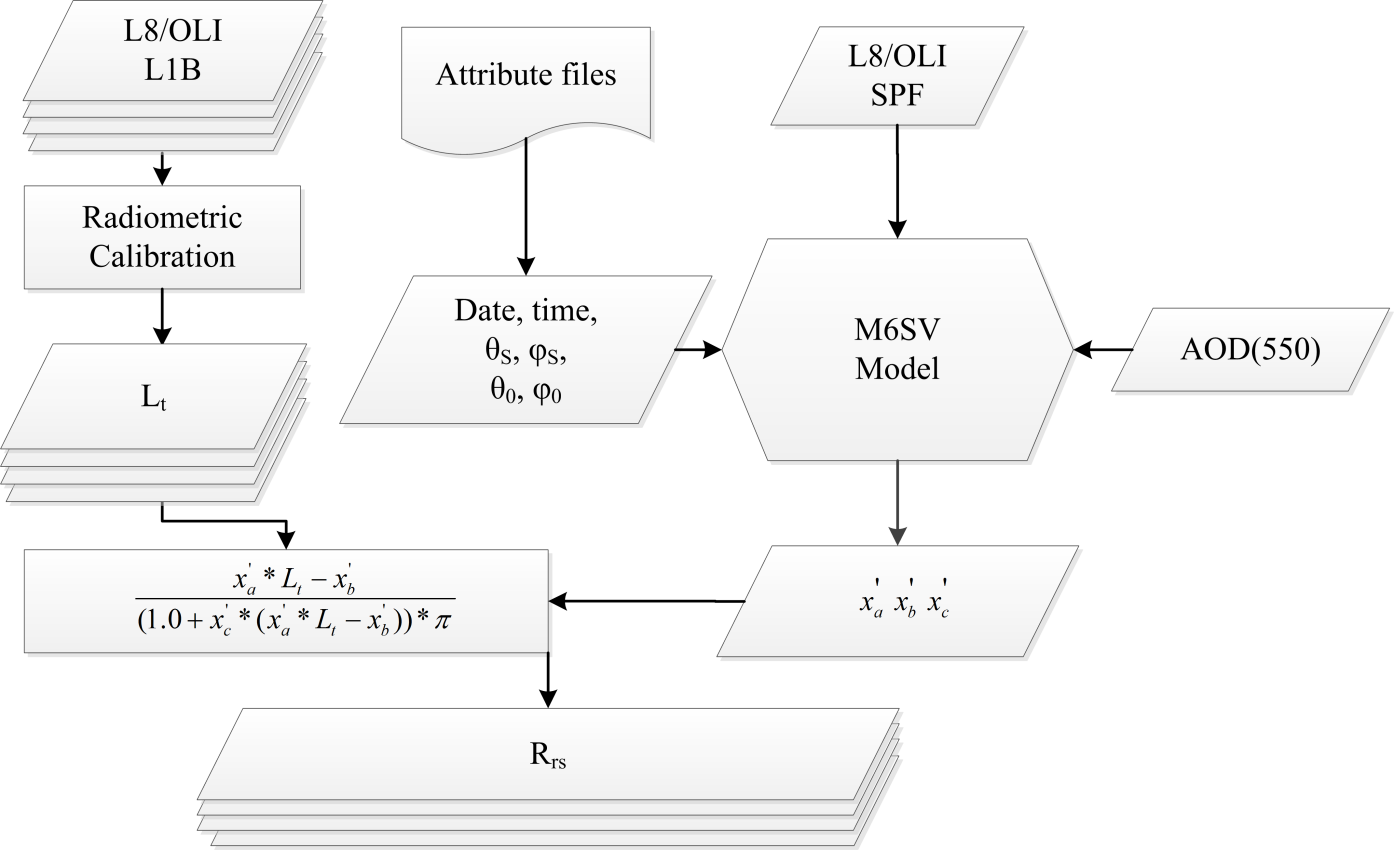
Flow chart of the M6SV atmospheric correction. L8/OLI L1B, the original L8/OLI L1B images; *L*_*t*_, the TOA radiance; Date, the image acquisition date; Time, the image acquisition time; θ_*s*_, the solar zenith angle; *φ*_*s*_, the solar azimuth angle; θ_*0*_, the sensor zenith angle; *φ*_*0*_, the sensor azimuth angle; L8/OLI SRF, the L8/OLI spectral response function; AOD550, the aerosol optical depth at 550 nm; *x*_*a*_′, *x*_*b*_′, and *x*_*c*_′, atmospheric correction parameters.

In addition to the modifications described above, the TOA radiance (*L*_*t*_) and synchronous image geometrical parameters must be prepared before atmospheric corrections can be performed. Fig 2 shows a flow chart of the atmospheric correction calculations in M6SV. The original L8/OLI L1B images are radiometrically calibrated to *L*_*t*_ using the gain and offset parameters extracted from the image metadata file for each band.

Information about θ_*s*_ (the solar zenith angle), *φ*_*s*_ (the solar azimuth angle), and the image acquisition date and time can be extracted from the image metadata file. Because L8/OLI views nearly vertically [34–35], θ_*0*_ (the sensor zenith angle) and *φ*_*0*_ (the sensor azimuth angle) were set to 0° in this study as per the methods of the United States Geological Survey (USGS) [36]. The mean AOD550 values were measured and calculated during *in situ* data collection. These input data indicate the most likely atmospheric conditions during image acquisition. The configuration parameters are detailed in Table 1. Using these data, M6SV calculates the atmospheric correction coefficients *x*_*a*_′, *x*_*b*_′, and *x*_*c*_′ of each band separately.

**Table 1:** M6SV configuration parameters for the L8/OLI image of Taihu Lake, China.

Item	Date	Time (UTC)	*θ*_s_ (°)	*φ*_s_ (°)	*θ*_0_ (°)	*φ*_0_ (°)	Atmospheric model	Aerosol model	AOD550
**Value**	10/26/2014	2:31:24	47.13	156.00	0	0	mid-latitude summer	continental	0.58

Date, the image acquisition date; Time, the image acquisition time; θ_*s*_, the solar zenith angle; *φ*_*s*_, the solar azimuth angle; θ_*0*_, the sensor zenith angle; *φ*_*0*_, the sensor azimuth angle; AOD550, the aerosol optical depth at 550 nm.

We ran VR-SWIR-F to obtain the *R*_*rs*_ of each band, considering the aerosol type fixed over the study area, using 1609 and 2201 nm for aerosol correction and a threshold on the Rayleigh-corrected reflectance in the 1609 nm for cloud and land masking. To obtain the *R*_*rs*_ of each band using the SD-SWIR model, we considered the aerosol type variable over the study area, chose 1609 and 2201 nm for aerosol correction, performed glint correction and cloud masking, and determined aerosol type per pixel.

### 2.6 Accuracy assessment

To evaluate the precision of the atmospheric correction, we compare the L8/OLI -derived *R*_*rs*_ values from the three different algorithms with those measured *in situ*. Synchronous image pixels are selected using the sampling site coordinates. *In situ* data from measurements carried out within ± 1 h of the L8/OLI overpass are chosen; a total of 13 synchropoints (Fig 1(B)) are used in the model comparison analysis.

The precision evaluation indices used for accuracy assessment include the mean ratio (MR), root mean square error (RMSE), and mean relative error (MRE), which are described by the following equations [37–38]:

$$\begin{array}{l} \mathrm{MR}=(\sum_{i=1}^{N}(R_{\mathrm{cal},i}/R_{\mathrm{mea},i}))/N,\\ \mathrm{RMSE}=\sqrt{\sum_{i=1}^{N}(R_{\mathrm{cal},i}-R_{\mathrm{mea},i}{)}^{2}/N},\\ \mathrm{MRE}=\mathrm{RMSE}/{\bar{R}}_{\mathrm{mea}}*100\%, \end{array}$$ (12)

where *R*_*cal*,*i*_ and *R*_*mea*,*i*_ refer to the *R*_*rs*_ estimated by the model and measured *in situ*, respectively; ${\bar{R}}_{\mathrm{mea}}$ is the average value of the *in situ* measurements; and *N* is the number of samples. MR is the mean ratio value between the model-derived and *in situ*-measured *R*_*rs*_ for each band, where MR values closer to 1 indicate that the model-derived value is closer to the *in situ* value and is therefore more accurate.

## Results and discussion

### 3.1 Visual inspection of atmospherically-corrected images

**Fig 3:**
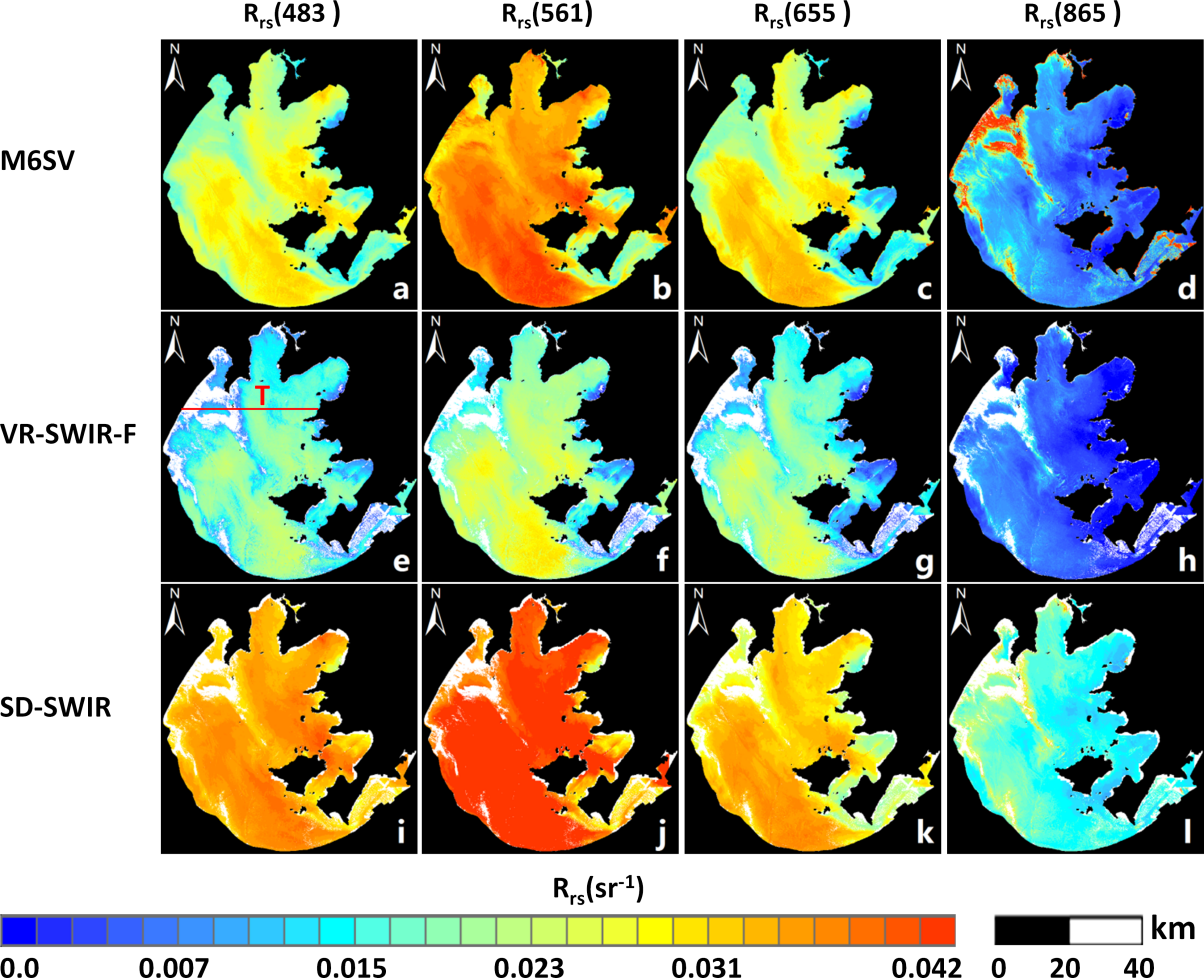
*R*_*rs*_ (*λ*) images derived by the M6SV, VR-SWIR-F and SD-SWIR models. Results for the atmospheric correction of an L8/OLI image (Taihu Lake, China, October 26, 2014) using the M6SV algorithm, for which (a) *R*_*rs*_(483), (b) *R*_*rs*_(561), (c) *R*_*rs*_(655), and (d) *R*_*rs*_(865) nm, the VR-SWIR-F algorithm, for which (e) *R*_*rs*_(483), (f) *R*_*rs*_(561), (g) *R*_*rs*_(655), and (h) *R*_*rs*_(865) nm, and the SD-SWIR algorithm, for which (i) *R*_*rs*_(483), (j) *R*_*rs*_(561), (k) *R*_*rs*_(655), and (l) *R*_*rs*_(865) nm.

The L8/OLI image of the research area was atmospherically corrected using, alternately, theM6SV, VR-SWIR-F and SD-SWIR models. *R*_*rs*_ images derived by the three models at wavelengths of 483, 561, 655, and 865 nm are shown in Fig 3. These *R*_*rs*_ images show overall *R*_*rs*_ spatial distributions for Taihu Lake.

Referring to Fig 3, the values of *R*_*rs*_ produced by the three models grow from 483 nm to 561 nm, and then decrease from 561 nm to 865 nm; this results primarily from the spectrally-dependent contributions of inherent optical properties (IOPs), constituent concentrations, and pure water absorption. The M6SV and SD-SWIR models produce larger *R*_*rs*_ values at each wavelength than does the VR-SWIR-F model. Further, *R*_*rs*_(561) values over Taihu Lake derived by Chen and Zhang [5, 39–40] are typically greater than 0.03 sr^-1^; the M6SV-derived *R*_*rs*_(561) and SD-SWIR-derived *R*_*rs*_(561) values are consistent with this research (Fig 3B and 3J).

Furthermore, the *R*_*rs*_ images derived by VR-SWIR-F and SD-SWIR are missing sections, as indicated by the white patches in Fig 3(E)–3(L); this is a result of the cloud masking operation [3], which defines Rayleigh-corrected reflectance values in Band 6 (centered at 1609 nm) greater than 0.0215 sr^-1^ as cloud pixels. However, in this case, the exceedingly high reflectance is caused not by clouds, but by an algal bloom event. Moreover, these sections are not missing from the M6SV results (Fig 3(A)–3(D)). Thus, the VR-SWIR-F and SD-SWIR models are invalid during the algal bloom event, while the M6SV model succeeds in obtaining valid atmospheric correction results.

For visible band, the water optical properties in southern Gonghu Bay show typical clear water characteristics. Whereas Taihu Lake is extremely turbid water, then the bottom radiance contributions to the *R*_*rs*_ values should be taken into consideration while the part of the lake is shallow. From Zhushan Bay to the center of the images, the M6SV *R*_*rs*_ values are low in the 483–655 nm bands but high in the 865 nm band. This phenomenon is caused by the algal bloom, for which the featured bands are 483 nm and 865 nm. The 483 nm band captures the strong absorption from algae, as the value of *R*_*rs*_(483) decreases with increased Chlorophyll-a during an algal bloom. However, low *R*_*rs*_(483) values can be also found over clear water due to the combined effects of backscattering and absorption. Thus, the high value of *R*_*rs*_(865) is also used to identify the bloom, as *R*_*rs*_(865) increases during bloom events in response to strong scattering by phytoplankton particles.

### 3.2 Methodological comparison

**Fig 4:**
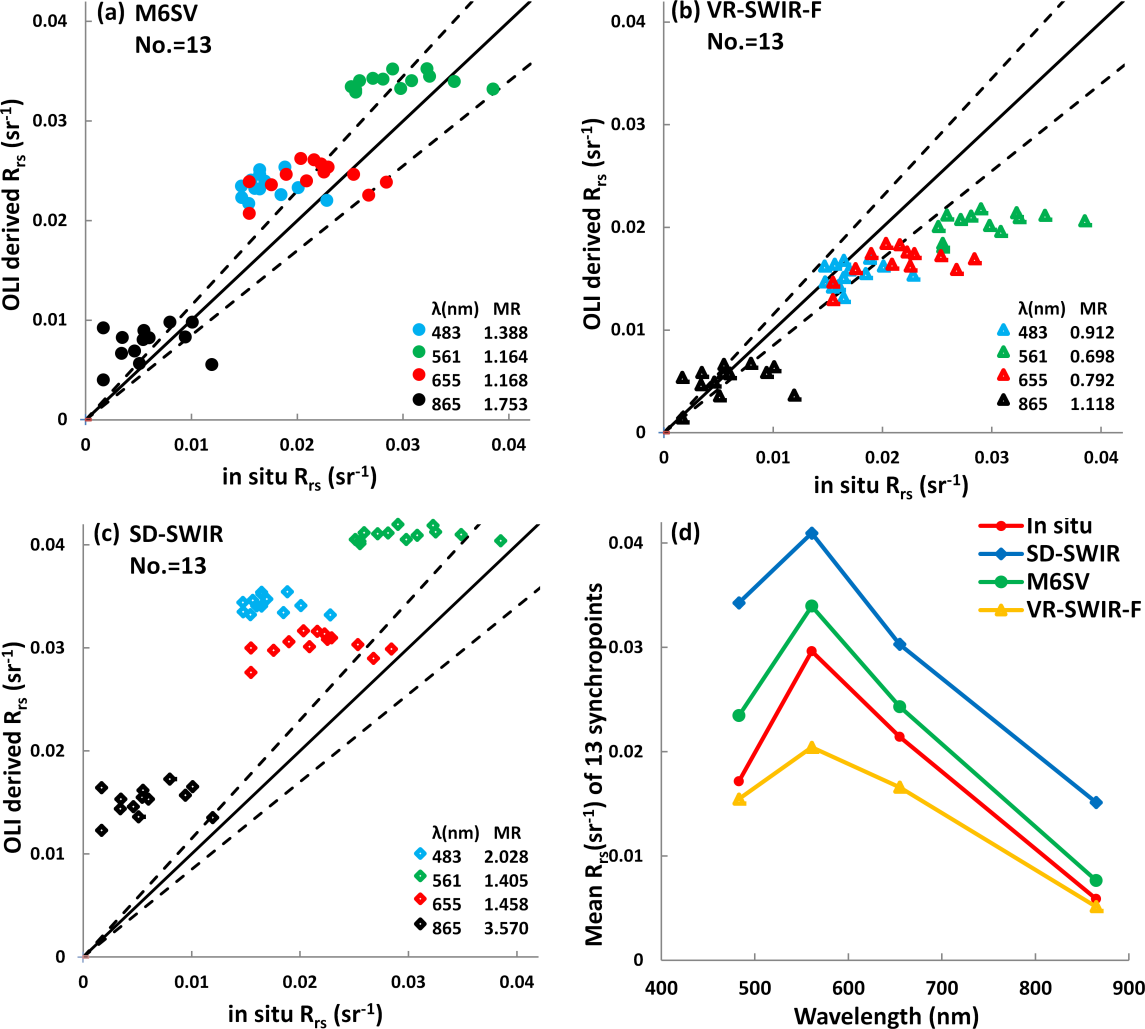
**L8/OLI *R***_***rs***_ **derived using the (a) M6SV, (b) VR-SWIR-F, and (c) SD-SWIR models plotted against *in situ* measurements from Taihu Lake at 13 synchropoints.** The solid line is a 1:1 line; the dashed lines represent ± 15% error around the 1:1 line. The mean ratio (MR) represents the ratio between the model-derived *R*_*rs*_ and *in situ*-measured *R*_*rs*_ for each band. (d) A comparison of the mean *R*_*rs*_ spectra from the three models and the *in situ* measurements at the 13 synchropoints.

In Fig 4, the M6SV-derived *R*_*rs*_, VR-SWIR-F-derived *R*_*rs*_, and SD-SWIR-derived *R*_*rs*_ are plotted against *in situ R*_*rs*_ measurements at 13 synchropoints. Fig 4 also shows a comparison between *in situ R*_*rs*_ measurements and the mean *R*_*rs*_ values at the 13 observation stations (Fig 1) derived using the three atmospheric correction models. The MR and number of synchropoints that fall within ± 15% error are shown in Table 2. Table 2 also reports the corresponding RMSE and MRE values for each model.

**Table 2:** Accuracy evaluation indices for the L8/OLI *R*_*rs*_ derived using the M6SV, VR-SWIR-F, and SD-SWIR models at each of the four spectral bands.

Index	Method	Band (nm)			
		483	561	655	865
MR	M6SV	1.388	**1.164**	**1.168**	1.753
	VR-SWIR-F	**0.912**	0.699	0.793	**1.118**
	SD-SWIR	2.028	1.405	1.458	3.570
# within ± 15% error	M6SV	1	**6**	**4**	3
	VR-SWIR-F	**9**	0	**4**	**4**
	SD-SWIR	0	1	2	1
RMSE (sr^-1^)	M6SV	0.007	**0.006**	**0.005**	**0.003**
	VR-SWIR-F	**0.003**	0.010	0.006	**0.003**
	SD-SWIR	0.017	0.012	0.010	0.010
MRE (%)	M6SV	39.688	**19.581**	**22.201**	61.595
	VR-SWIR-F	**16.447**	33.360	27.546	**51.798**
	SD-SWIR	100.752	40.430	44.938	165.965

Fig 4 and Table 2 indicate that the M6SV model offers improved atmospheric correction performance for the L8/OLI image compared to the SD-SWIR model. The M6SV model under-corrects the image, while the VR-SWIR-F model over-corrects the image.

In Fig 4(A)–4(C), the relationship between the data points and the 1:1 line represents the performance of the corresponding model. In order to contextualize the data, ± 15% error lines are given about the 1:1 line; when the data produced by a specific model fall between these dotted lines, the atmospheric correction error of that model is within ± 15%. The distribution of the M6SV data shows that M6SV outperforms (i.e., features lower error than) SD-SWIR at all bands and VR-SWIR-F at 561 nm, while possessing error similar to VR-SWIR-F at 655 and 865 nm.

Fig 4(D) shows that the *R*_*rs*_ spectra retrieved by all three models fall within the same order of magnitude. However, all of the SD-SWIR *R*_*rs*_ values are larger than the M6SV *R*_*rs*_ values. Additionally, when comparing mean values of *R*_*rs*_ produced by M6SV and VR-SWIR-F (Fig 4(D)), it is clear that M6SV performs well at 561 and 655 nm, while VR-SWIR-F performs well at 483 and 865 nm; the evaluation indices in Table 2 support this conclusion. The MR and the MRE values show that the M6SV model performs best at 561 and 655 nm, while the VR-SWIR-F model performs best at 483 and 865 nm. The number of data points within ± 15% error and the RMSE results show that the M6SV model performances best at 561 nm, while the VR-SWIR-F model performs best at 483 nm; the two methods perform comparably at 655 and 865 nm.

For M6SV, the RMSE decreases from 483 to 865 nm, indicating that the precision of the derived result increases. The MRE decreases from 483 to 561 nm, then increases from 561 to 865 nm, and is less than 22.2% at 561 and 655 nm; these values indicate that the M6SV model performed well in the retrieval of *R*_*rs*_ values for Taihu Lake at 561 and 655 nm. At 865 nm, the reflectance signal in the NIR band is exceedingly small, which may explain why the MRE is greater than 50%.

Typically, in turbid water, signals in the blue band (483 nm) are depressed due to the characteristically high absorption by Chlorophyll-a (C*a*) and CDOM in this spectral region. For this reason, the green, red, and NIR bands, rather than the blue band, are used for C*a* and Total Suspended Sediment (TSS) retrievals [17, 41–43]. Therefore, results from the M6SV model have practical potential for the quantification of quality in extremely turbid waters.

### 3.3 Sample comparisons

**Fig 5:**
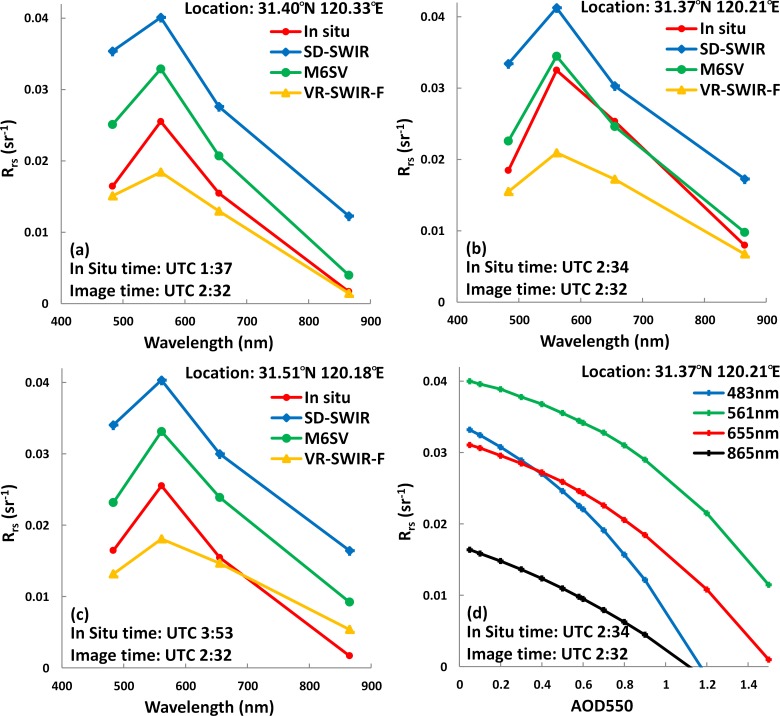
**(a-c) Examples of validation comparisons between model-derived *R***_***rs***_ **and *in situ* measurements from Taihu Lake matched sampling points.** The latitudes and longitudes of the matched sampling points, times of image and *in situ* spectra acquisition, and models used to derive *R*_*rs*_ are listed. (d) Values of M6SV-derived *R*_*rs*_ at 4 bands changing with the value of AOD550.

Three example validation comparisons are shown in Fig 5. The three datasets were obtained at different locations and different times. Fig 5(A)–5(C) show *R*_*rs*_ data collected at the locations indicated in Fig 1(B). The collection times of 01:37, 02:34, and 03:53 (UTC) correspond to approximately 1 hour before, the time of, and 1 hour after the L8/OLI overpass. The L8/OLI-measured *R*_*rs*_ spectral data in Fig 5 were derived by averaging a 1 × 1 pixel area surrounding the *in situ* data site.

The results in Fig 5 further support the conclusions reached above. First, M6SV shows better atmospheric correction performance than SD-SWIR at all bands. The M6SV model also produces better spectral shapes than does the VR-SWIR-F model. It is also apparent that M6SV performs better than VR-SWIR-F at the time of the L8/OLI overpass. M6SV performance appears to be sensitive to timing, which may be caused by a temporal mismatch between satellite and *in situ* data. Increased *in situ* data time resolution may improve the M6SV validation results.

Considering relative stability of other M6SV configuration parameters on time scale, the sensitivity of AOD550 to the M6SV are analyzed and discussed here. The M6SV parameters in Table 1 were kept unchanged except for AOD550 values. The AOD550 values are 0.1, 0.2, 0.3, 0.4, 0.5, 0.58, 0.6, 0.7, 0.8, 0.9, 1.2 and 1.5, respectively, for atmospheric correction. For sample (31.37° N, 120.21° E), *R*_*rs*_ values derived by the M6SV at wavelengths of 483, 561, 655, and 865 nm, changing with the AOD550 values, are shown in Fig 5(D). The influence trend in atmospheric correction results of M6SV is basically consistent. That is, the *R*_*rs*_ values decrease linearly with the increase of AOD550 for AOD550 less than 0.6. When AOD550 greater than 0.6, the rate of decrease in *R*_*rs*_ value is increasing with the increase of AOD550. The influence of AOD550 on *R*_*rs*_ decreases with the increase of wavelength. The blue band is the most affected while the near-infrared band is largely unaffected. It may be because attenuation of scattering appears when the wavelength is greater than the aerosol diameter.

### 3.4 Transect analysis

**Fig 6:**
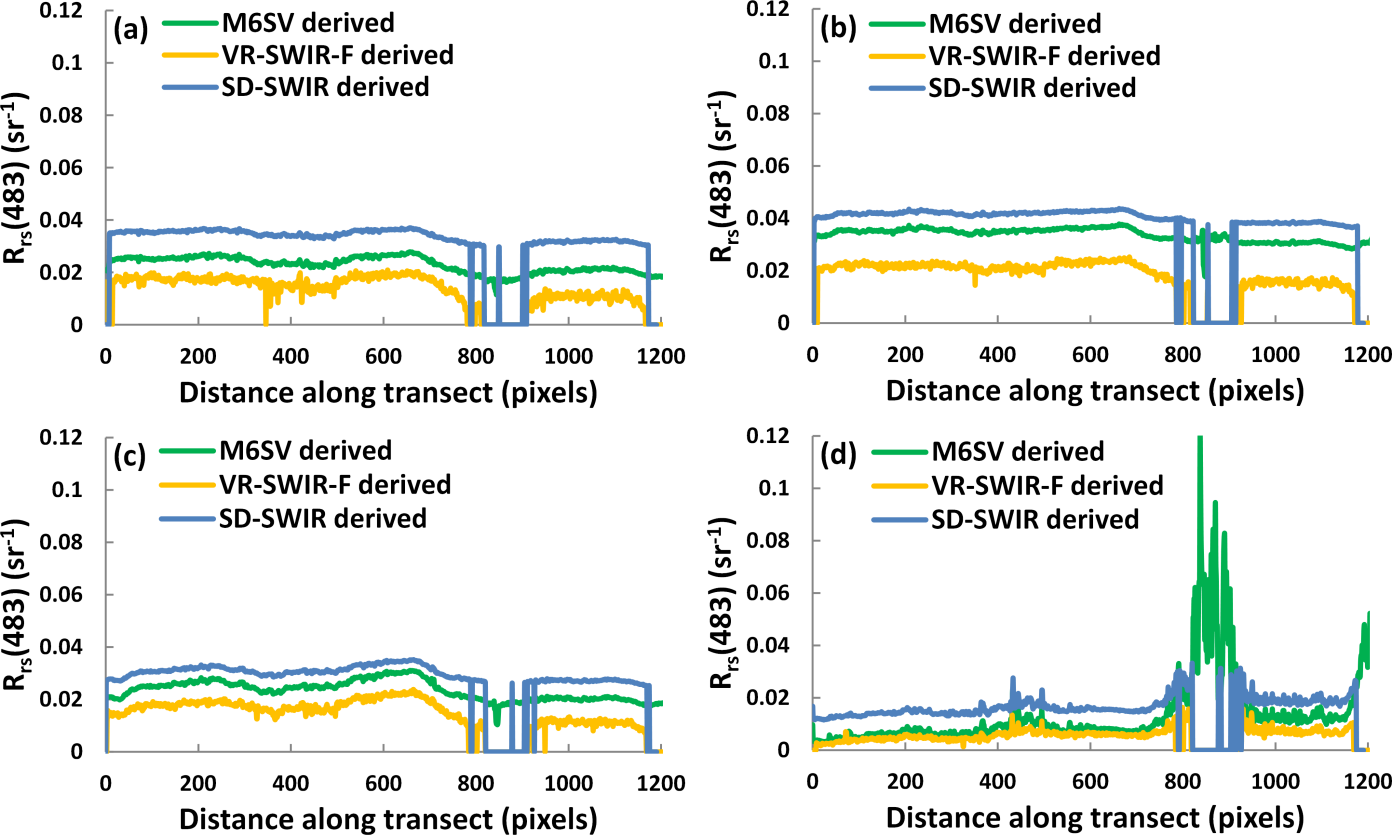
*R*_*rs*_ (*λ*) extracted from pixels along the transect ‘T’ in Fig 3(E). *R*_*rs*_ (*λ*) were derived using the (green) M6SV, (yellow) VR-SWIR-F and (blue) SD-SWIR models at wavelengths of (a) 483, (b) 561, (c) 655, and (d) 865 nm.

*R*_*rs*_ spatial distributions derived by M6SV, VR-SWIR-F and SD-SWIR are exhibited in Fig 3. To further demonstrate the differences among the three models, the *R*_*rs*_ values of pixels lying along the transect ‘T’ (Fig 3(E)) are extracted at wavelengths of 483, 561, 655, and 865 nm. These profiles are shown in Fig 6, where the different colors represent the three models used. From left to right, the x-axis pixel values correspond to Gonghu Bay, then Meilang Bay, and finally Zhushan Bay; the selected transect covers turbid waters and the algal bloom area.

In Fig 6, the spectral shapes produced by the three models are generally consistent. The *R*_*rs*_ values derived by SD-SWIR are larger than those derived by M6SV while the *R*_*rs*_ values derived by M6SV are larger than those derived by VR-SWIR-F. With the exception of pixels over the algal bloom, the degree of turbidity along the transect is similar because the *R*_*rs*_ profiles are similar. The 483 and 865 nm bands are most useful for the algal bloom pixels, as explained previously. Thus, for pixels over the algal bloom, the *R*_*rs*_ values derived by M6SV are small at 483 nm and large at 865 nm, while the *R*_*rs*_ values derived by VR-SWIR-F and SD-SWIR are NaNs (Not-a-Number), as these pixels were masked by the algorithms. For pixels over low algal bloom concentrations, the pixels are not masked by the VR-SWIR-F and SD-SWIR model; for such pixels, the *R*_*rs*_ values derived by the two models are small at 483 nm and slightly larger at 865 nm.

### 3.5 Density-sliced scatterplots

**Fig 7:**
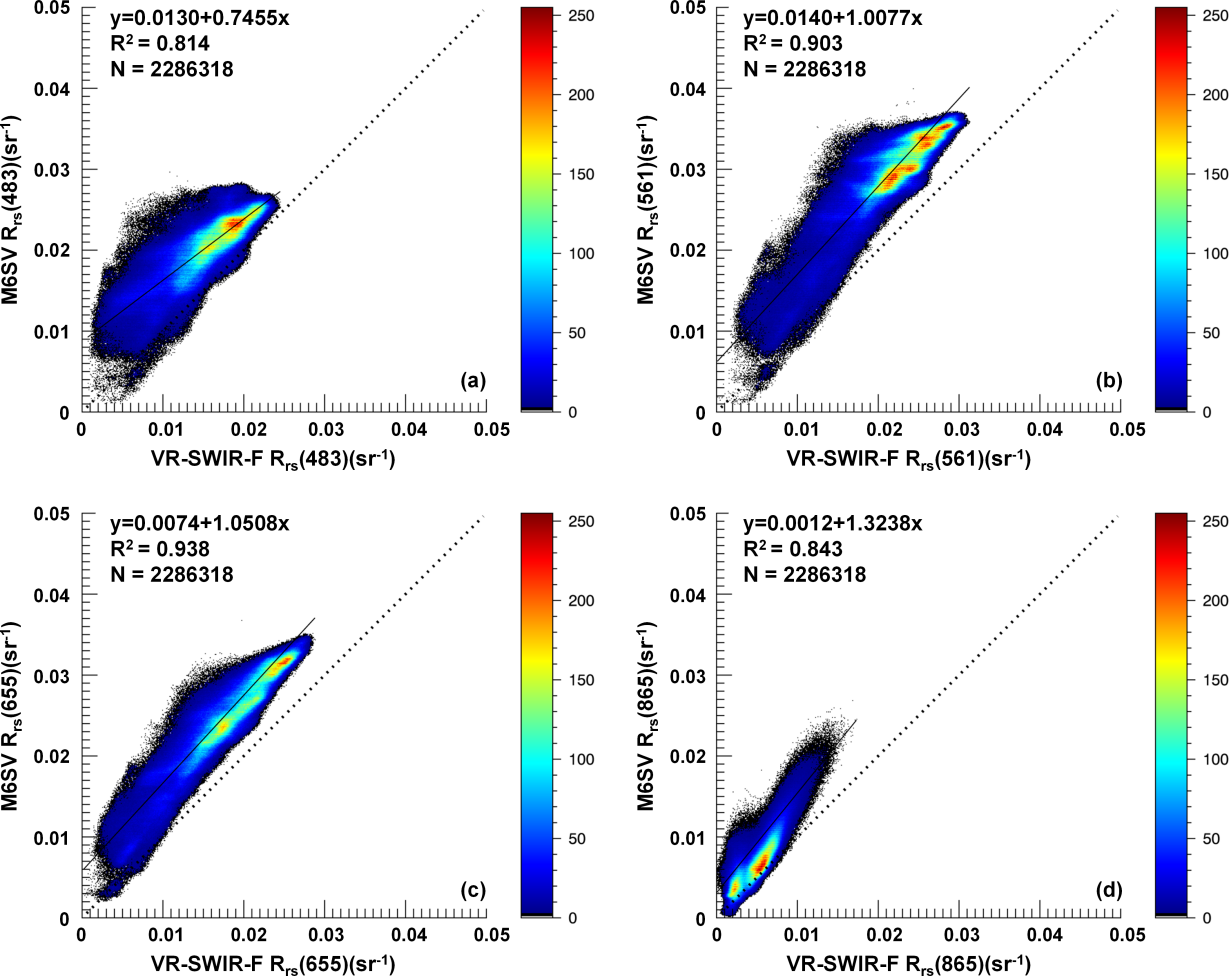
***R***_***rs***_ **(*λ*) density-sliced scatterplots at wavelengths of (a) 483, (b) 561, (c) 655, and (d) 865 nm.** The plots show the relationship between the M6SV and VR-SWIR-F models for a L8/OLI image taken over Taihu Lake, China, on October 26, 2014.

In order to compare the performance of the two models for each pixel over the entire lake area, a L8/OLI image taken over Taihu Lake on October 26, 2014 was processed using both the M6SV and VR-SWIR-F models. Density-sliced scatterplots of the M6SV estimates versus the VR-SWIR-F estimates at wavelengths of 483, 561, 655, and 865 nm are shown in Fig 7. R^2^ values for the two models range from 0.814 at 483 nm to 0.938 at 655 nm, indicating high correlations. The *R*_*rs*_ values derived by M6SV are larger than those derived by VR-SWIR-F for each band over the entire lake area; this comparison of *R*_*rs*_ values and distribution trends supports the validation and transect results.

## Conclusions

In this study, a modified 6SV atmospheric correction algorithm (M6SV) is developed to implement a skylight correction. This algorithm is then applied to the atmospheric correction of a L8/OLI image over the extremely turbid inland waters of Taihu Lake in China. To validate the algorithm, M6SV-derived, SD-SWIR-derived, and VR-SWIR-F-derived *R*_*rs*_ values are calculated and compared with the *in situ* measured reflectance. These comparisons show that the M6SV method improves L8/OLI atmospheric correction performance over the SD-SWIR method at all bands and over the VR-SWIR-F method at 561 and 655 nm. The M6SV MRE values at 561 and 655 nm are less than 22.2%, indicating that the model works well. With the derived *R*_*rs*_, we can retrieve water quality parameters (such as C*a* and TSS) that can be used to monitor the optical and biological properties of inland waters and establish a real-time, wide-ranging, and long-term water quality time series.

## Supporting information

S1 Table Full dataset for the *in situ* measurements, along with geographical coordinates for the sampling stations. (XLSX)
